# An epidemiological survey of tumour or tumour like conditions in the scapula and periscapular region

**DOI:** 10.1051/sicotj/2016023

**Published:** 2016-10-14

**Authors:** Zeeshan Khan, Adam M. Gerrish, Robert J. Grimer

**Affiliations:** 1 The Bone Tumour Unit, The Royal Orthopaedic Hospital Bristol Road South, Northfield Birmingham B31 2AP UK

**Keywords:** Scapula, Periscapular, Lesions, Tumours, Sarcoma

## Abstract

*Introduction*: The scapula is not an uncommon site for bone and soft tissue tumours and can be difficult to delineate on examination. Furthermore, these lesions can be potentially challenging to biopsy due to its close anatomical relationship with important structures. We present an epidemiological survey of all the scapular and periscapular lesions presenting to our institution.

*Methodology*: This was a retrospective study with data obtained from a prospectively held electronic database over a 30-year period. Demographic and clinical data was obtained and various subgroup analyses were performed.

*Results*: A total of 418 scapular lesions were included in the study where 132 lesions were found to be of soft tissue origin and 286 were osseous. Fifty-eight percent (*n* = 241) of all these lesions were malignant, of which 47% (*n* = 113) were primary sarcomas. The commonest malignant lesions were bone sarcomas (*n* = 96) followed by metastases (*n* = 88). The commonest primary bone sarcoma was chondrosarcoma (45%), whereas the commonest soft tissue sarcoma was high grade undifferentiated pleomorphic sarcoma (18%). The most common benign osseous and soft tissue lesions were osteochondroma (70%) and lipoma (26%), respectively. We noted that the incidence of malignancy increased with increasing age, however, the incidence of primary bone sarcomas was fairly consistent across different age groups.

*Conclusion*: Based on our findings we recommend that suspicious lesions arising from the scapula should be dealt with in a specialist sarcoma unit with involvement of a multidisciplinary team to offer appropriate management and advice for optimum outcome.

## Introduction

The shoulder girdle is comprised of the proximal humerus, scapula, clavicle and the surrounding soft tissues and is the third most common site for bone and soft tissue tumours [1, 2]. In a report of 1853 cases of bone cancer, 3.6% (*n* = 66) were found to originate from the scapula [3]. The scapula provides attachment to 17 muscles and is also affected by soft tissue sarcomas (STS) which can be difficult to delineate from bony pathology on examination. A further wide array of pathological conditions including metastatic deposits, haematological malignancies and non-malignant pathologies also present as suspicious scapula lesions.

Previous studies have shown that primary bone tumours of the scapula are more likely to be malignant than benign and the three most common subtypes of malignant bone tumours are osteosarcoma, chondrosarcoma and Ewing’s sarcoma [2, 5]. Although Ewing’s sarcoma is the second most common malignant bone tumour of children and young adults, it occurs rarely in the scapula [4]. To our knowledge, very few cases of Ewing’s sarcoma of scapula have been reported in the literature so far [6, 7].

The majority of the scapula forms by intramembranous ossification. Ossification of the body and the spine of the scapula occurs before birth, but ossification of the coracoid process, glenoid, acromion, vertebral border and inferior angle takes place throughout childhood, with the two ossification centres unifying at approximately 15 years of age.

The scapula presents itself as a challenging site to perform biopsies due to a number of factors. The most important are the neurovascular bundles and it is also important to avoid contamination of the non-involved muscular planes and injury to the chest wall [2]. Early diagnosis of scapula lesions is critical in improving outcomes, but this can be challenging due to the infrequency with which these conditions are encountered and the number of different pathologies affecting this area.

Due to the relative infrequency of these lesions, we set out to review all the lesions referred to our national sarcoma unit arising from the scapula or periscapular tissue and look at various demographic factors in association with these lesions. We aim to provide some diagnostic guidance when these factors are considered but management in multidisciplinary team setting and biopsy still remain the gold standard. This current study does not include clinical outcomes of these lesions.

## Methodology

A retrospective review of a prospectively held oncology database at our institution was conducted and all patients presenting with a suspicious scapula lesion over a period of 30 years (1985–2015) were included in this study. Data including patient demographics, histological diagnosis, site and laterality was collected and further subgroup analysis was performed for different diagnosis and age groups (in decades).

## Results

A total of 418 patients were found to be eligible for inclusion in the study. All these patients had been referred to us for a “suspicious” lesion in or around the scapula and were discussed in our multidisciplinary team meeting with a review of their clinical and radiological pictures. The median age for the whole cohort was 47 years (range 0–88), and 60% (*n* = 249) of the study population were male. Fifty-two percent (*n* = 216) of the patients had involvement of the right-sided scapula.

One hundred and thirty-two (32%) lesions were found to be of soft tissue origin and 286 (68%) were osseous. Overall, 40% (*n* = 169) of lesions were benign, 57% (*n* = 240) were malignant and 2% (*n* = 9) of the lesions were reported as being borderline (Table 1). Females had a slightly higher percentage of malignant lesions than males (62% vs. 55%).

**Table 1.** Distribution of different lesions of the scapula in various age groups.

Diagnosis	0–9 years	10–19 years	20–29 years	30–39 years	40–49 years	50–59 years	60–69 years	70–79 years	80–89 years	Total
Benign bone tumour	23	22	25	17	6	3	3	2	1	102
Benign soft tissue tumour	1	5	2	6	9	11	9	7	0	50
Bone sarcoma	7	18	12	9	11	14	12	11	2	96
Haematological malignancy	0	1	1	6	6	8	9	7	2	40
Metastasis	0	0	0	4	12	11	27	23	11	88
Non-oncological diagnosis	1	4	3	1	6	3	5	1	1	25
Soft tissue sarcomas	1	0	3	1	4	1	4	2	1	17
Total	33	50	46	44	54	51	69	53	18	418

Eighty-five percent (*n* = 96) of all sarcomas were osseous in origin and 15% (*n* = 17) originated from soft tissues. Soft tissue tumours were more likely to be benign (*n* = 50) than malignant (*n* = 17) whereas the number of benign and primary malignant osseous lesions was similar (102 vs. 96, respectively).

## Subgroup analysis

### Benign lesions

**Figure 1.**
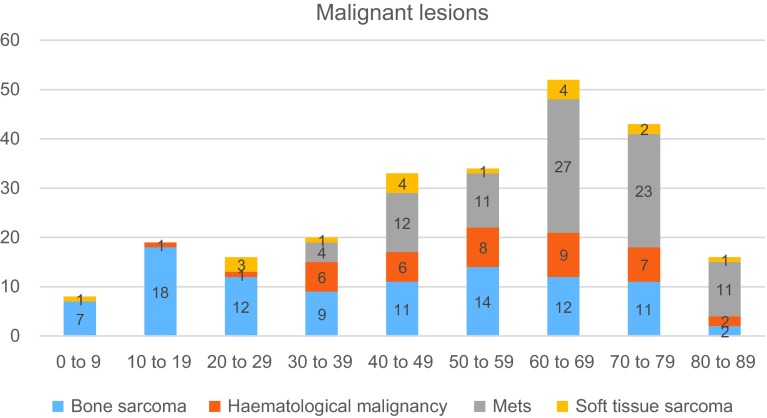
The subtypes of malignant lesions of the scapula.

Forty-two percent (*n* = 177) of all the scapular lesions were benign of which 58% (*n* = 102) were osseous and 42% (*n* = 75) were soft tissue in origin (Figure 1). Osteochondroma was the commonest benign osseous tumour (70%, *n* = 71) whereas the commonest soft tissue tumours were lipoma, elastofibroma and fibromatosis (Tables 2–4). Non-oncological diagnoses made up 14% (*n* = 25) of the benign lesions.

**Table 2.** Most common benign lesions according to age.

Age category	Most common benign lesion diagnosis
0–9	Osteochondroma (64%, *n* = 16)
10–19	Osteochondroma (48%, *n* = 15)
20–29	Osteochondroma (60%, *n* = 18)
30–39	Osteochondroma (50%, *n* = 12)
40–49	Non-oncological (29%, *n* = 6)
50–59	Non-oncological (18%, *n* = 3), lipoma (18%, *n* = 3)
60–69	Non-oncological (24%, *n* = 4), elastofibroma (24%, *n* = 4)
70–79	Lipoma (30%, *n* = 3), elastofibroma (30%, *n* = 3)
80–89	Osteochondroma (50%, *n* = 1), non-oncological (50%, *n* = 1)

**Table 3.** Commonest benign bone tumours of the scapula.

Subtype of benign bone tumour	Number of cases
Osteochondroma	71
Aneurysmal bone cyst	7
Eosinophilic granuloma	6
Fibrous dysplasia	3
Arteriovenous malformation	2
Giant cell tumour of bone	2
Osteoid osteoma	2
Others	9
Total	102

**Table 4.** Commonest benign soft tissue tumours of the scapula.

Subtype of benign soft tissue tumour	Number of cases
Lipoma	13
Elastofibroma	10
Fibromatosis	7
Enchondroma	4
Intramuscular lipoma	4
Ganglion	2
Others	10
Total	50

### Malignant lesions

Fifty-eight percent (*n* = 241) of all the lesions were malignant of which 113 were primary sarcomas, 88 metastasis and 40 were haematological malignancies (Figure 1 and Table 1).

**Figure 2.**
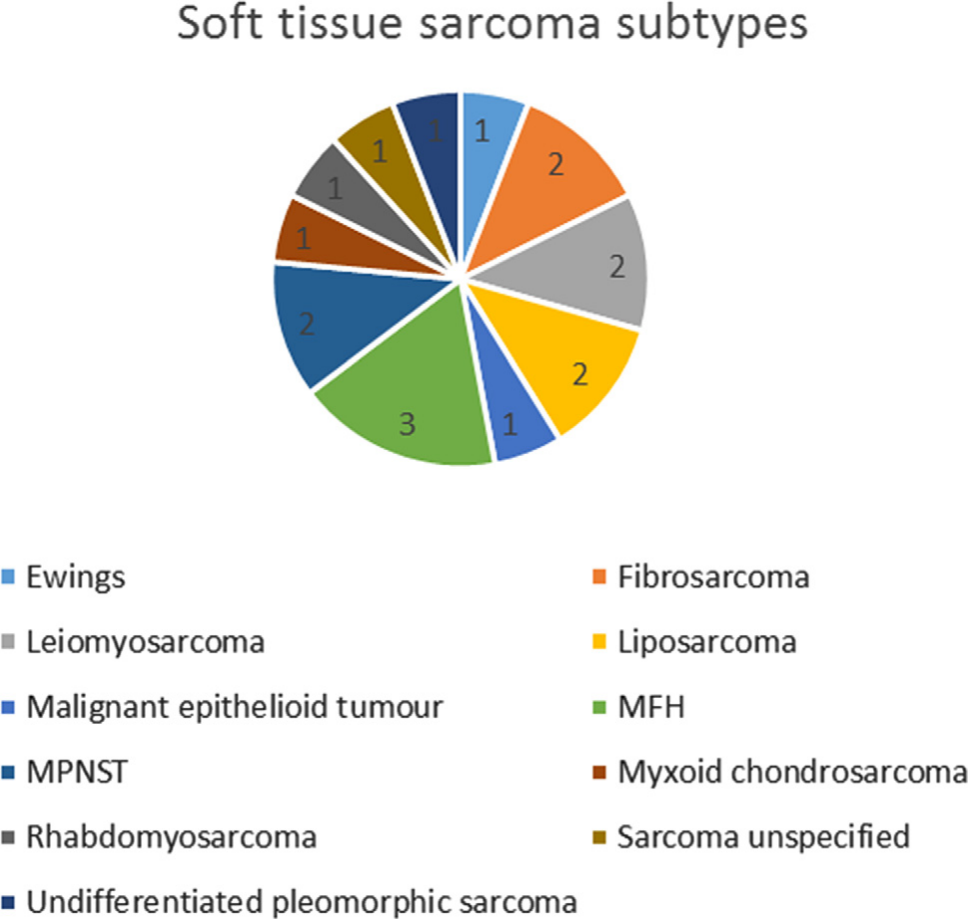
The subtypes of soft tissue sarcomas of the scapula.

Chondrosarcoma was the most common primary malignant bone tumour (45%, *n* = 43) followed by Ewing’s sarcoma (25%, *n* = 24) (Table 5). Of the 17 STS, a wide range of histological diagnosis was reported and the commonest reported tumour was malignant fibrous histiocytoma, now called high grade undifferentiated pleomorphic sarcoma (18%, *n* = 3) (Figure 2).

**Table 5.** Most common bone sarcomas according to age.

Age category	Most common bone sarcoma diagnosis
0–9	Ewing’s (100%, *n* = 7)
10–19	Ewing’s (67%, *n* = 12)
20–29	Chondrosarcoma (75%, *n* = 8)
30–39	Chondrosarcoma (78%, *n* = 7)
40–49	Chondrosarcoma (82%, *n* = 9)
50–59	Chondrosarcoma (64%, *n* = 9)
60–69	Chondrosarcoma (42%, *n* = 5)
70–79	Chondrosarcoma (27%, *n* = 3), sarcoma unspecified (27%, *n* = 3)
80–89	Chondrosarcoma (50%, *n* = 1), secondary osteosarcoma (50%, *n* = 1)

Lungs, kidneys and adenocarcinoma of unknown origin, respectively, were the commonest primary sites for metastasis. Forty-three percent (*n* = 17) of all the haematological malignancies in our series were non-Hodgkin’s lymphomas followed by plasmacytomas in 28% (*n* = 11) of the cases.

### Diagnosis according to age

The commonest age for presentation of any lesion in scapula was in the 6th decade (*n* = 69) (Table 1). In the first decade of life the percentage of benign conditions was 76% (*n* = 25) but this falls to just 11% (*n* = 2) by the 9th decade. Over the first four decades of life 62% (*n* = 104) of lesions were benign, and were predominantly benign bone tumours (*n* = 87) of which 70% (*n* = 61) were osteochondromas (Table 2). Eighty-six percent (*n* = 61) of all osteochondromas were found in the first four decades of life. Between the 5th and 9th decade just 25% (*n* = 60) of lesions were found to be benign.

Between the 5th and 8th decade benign soft tissue tumours were the commonest benign lesion (55%, *n* = 36). The most common subdiagnosis between the 5th and 9th decade is non-oncological diagnosis (Table 2).

The percentage of malignant conditions steadily increases with increasing age (Figure 1). The number of malignancies increases from the 1st decade (*n* = 8) to a peak in the 7th decade (*n* = 49). Primary bone sarcomas were distributed almost equally across the age groups (median = 11, IQ range = 3) however the subdiagnosis of bone sarcoma varies by age. In the first four decades the most predominant type of malignant lesion is bone sarcoma (73%, *n* = 46). Chondrosarcoma mainly presented in the 3rd–6th decades and accounted for 61% (*n* = 43) of bone sarcomas in patients above 20-year age group. Ewing’s sarcoma accounted for 25% (*n* = 24) of bone sarcomas and 79% (*n* = 19) of these were found in the first two decades of life (Table 5).

Metastases first appeared in patients aged over 30 years and were the most predominant type of lesion in the 7th–9th decades of life (44%, *n* = 61). This shows a trend from sarcomas towards metastasis with increasing age. There were a total of 40 haematological malignancies, with 60% (*n* = 24) in the 6th–8th decades. Soft tissue sarcomas only made up 7% (*n* = 17) of the malignant lesions and do not appear to affect any single age group predominantly (median = 1, IQ range = 2) (Figure 1).

## Discussion

The rarity of suspicious lesions of the scapula along with its variable vasculature and difficulty to biopsy increases the challenges associated with the diagnostic workup. There is paucity of published literature on these lesions but a smaller series of 68 lesions found more than one third to be osteochondromas, a quarter to be chondrosarcomas and one third to be a number of other benign and malignant entities [8].

Fifty-seven percent (*n* = 240) of all the lesions in our case series were found to be malignant, consistent with previous research showing that primary bone tumours of the scapula are more likely to be malignant than benign [4]. The percentage of malignant conditions steadily increases with increasing age from 24% (*n* = 8) in the first decade of life to 89% (*n* = 16) in the 9th decade. This demonstrates an increased risk of lesions being malignant with increased age.

**Figure 3.**
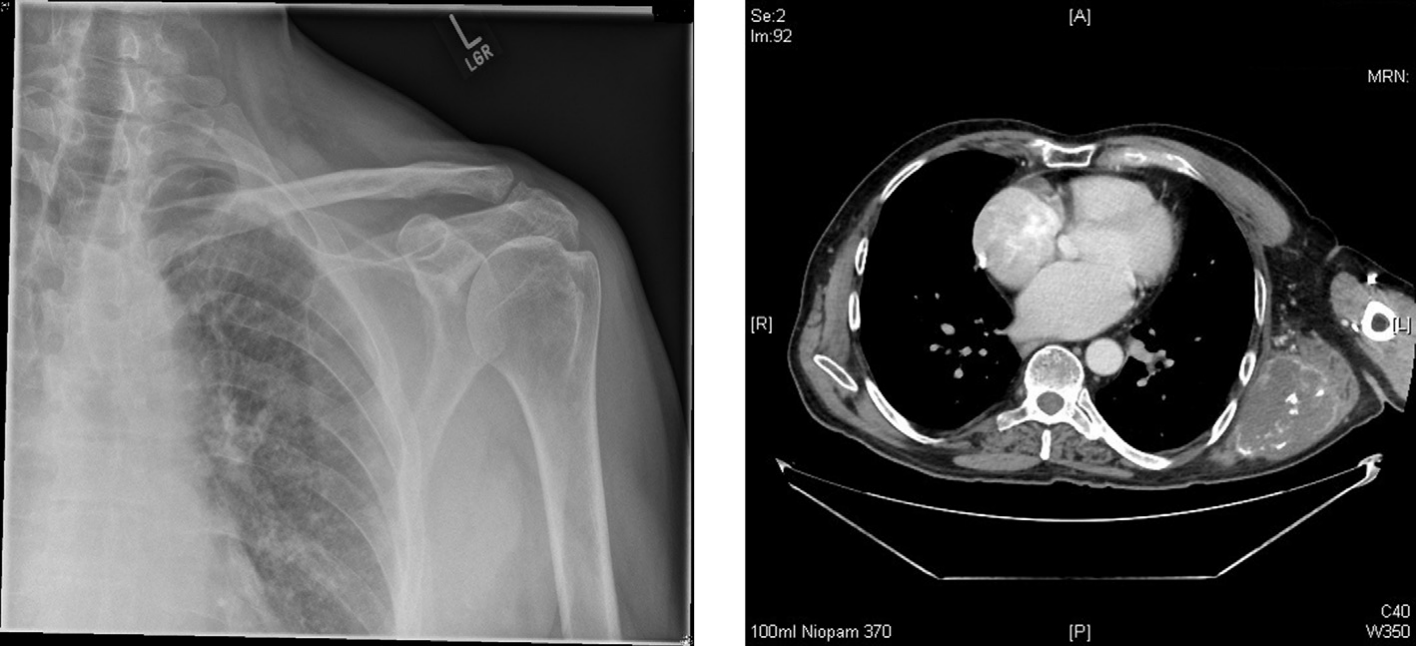
Plain radiograph of left shoulder girdle only showing some soft tissue swelling around the lateral border of the scapula whereas the CT scan demonstrates a destructive lesion consistent with chondrosarcoma.

**Figure 4.**
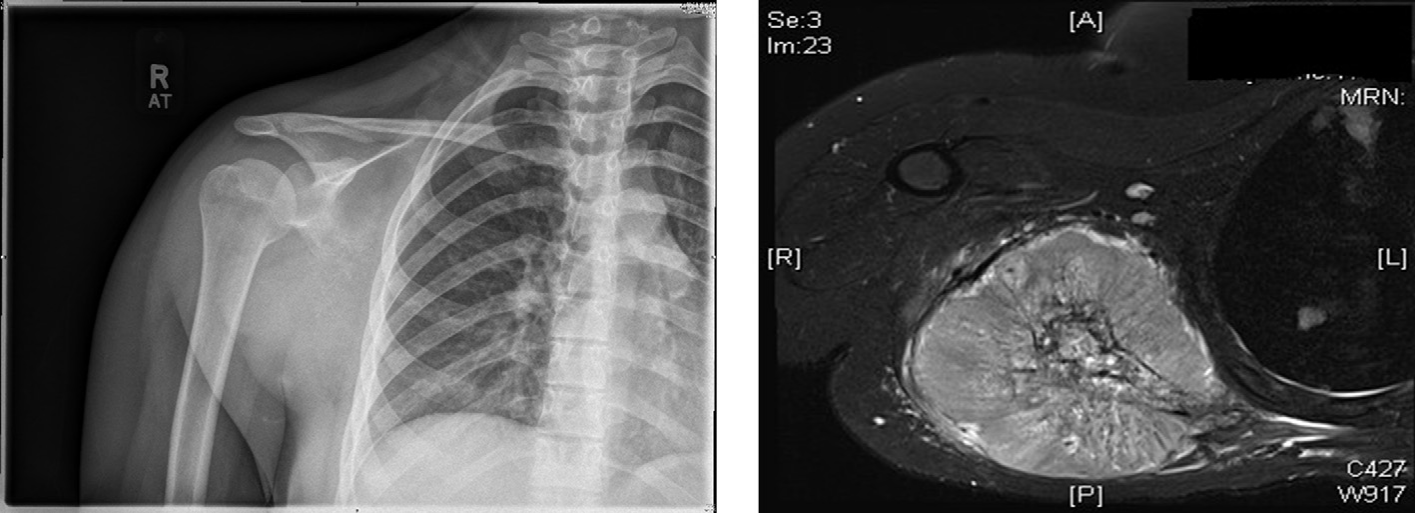
Plain radiograph demonstrating a destructive lesion of the right scapula confirmed with axial view of MRI scan showing a large soft tissue component to the scapular lesions consistent with Ewing’s sarcoma.

Of the 96 bone sarcomas arising from the scapula, the three most common subtypes were found to be chondrosarcoma (*n* = 43), Ewing’s sarcoma (*n* = 24) and osteosarcoma (*n* = 19) which is similar to previously reported studies (Figures 3 and 4). The incidence of osteosarcomas however shows a bimodal distribution with peaks in the 2nd decade of life (*n* = 4) as well as in the 6th–7th decades (*n* = 9). Primary osteosarcomas account for the early age cases but osteosarcomas in the later ages are secondary to Paget’s disease and previous radiotherapy [2].

Chondrosarcoma is the second most common primary sarcoma of bone [9]. Only 10% (*n* = 43) of our cases were found to be chondrosarcomas which is a lower proportion than that found in a study by Brtková et al. [8]. In our series we found that chondrosarcomas were common in the 3rd–6th decades, whereas other studies have found the 4th–7th decades as commonest [8]. This however, is not significantly different as we expect chondrosarcomas to be common in any age after 30s.

Seventy-nine percent (*n* = 19) of Ewing’s sarcomas were found in the first two decades of life which is as expected and reported the same by other studies [2].

The distribution of Ewing’s sarcoma was almost the same in different genders in our series whereas another study has reported this to be 1.6:1 (male vs. female) [10]. Two cases of Ewing’s sarcoma were diagnosed in patients over the age of 30 which is a rare occurrence [2].

**Figure 5.**
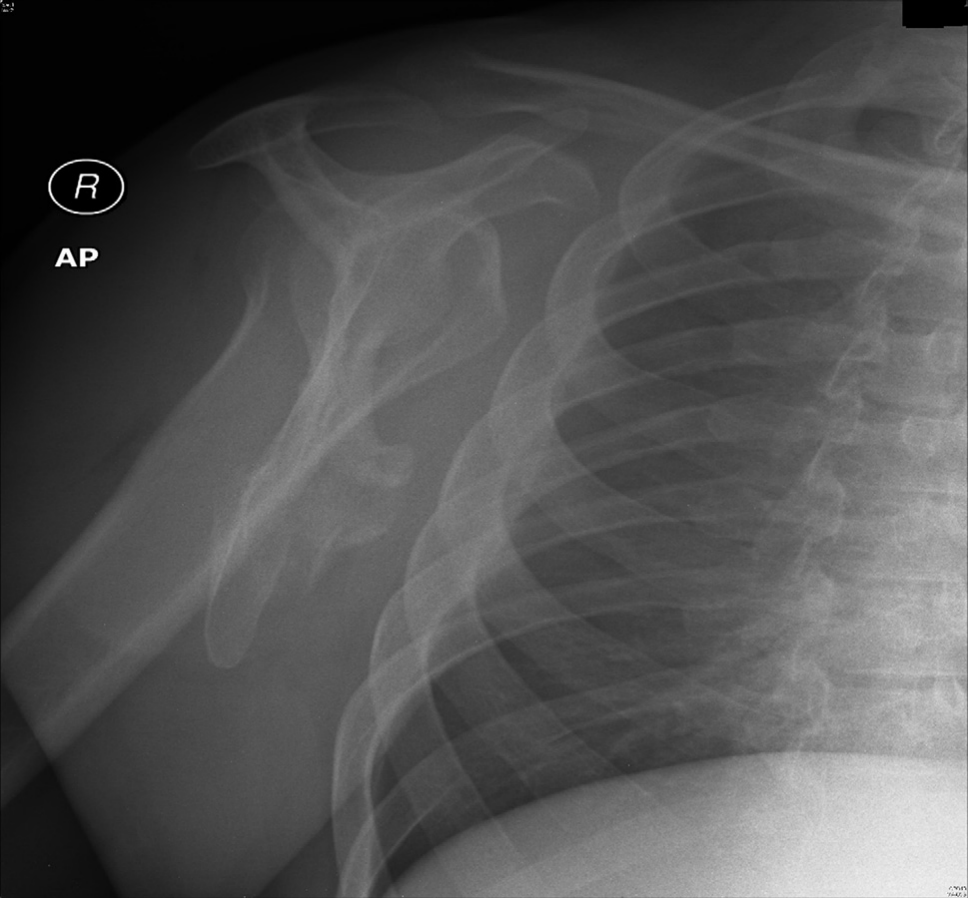
Lateral view of plain radiograph of right scapula demonstrating a broad based pedunculated osteochondroma.

We also found that the most common soft tissue sarcomas were high grade undifferentiated pleomorphic sarcoma, previously called malignant fibrous histiocytoma (MFH), liposarcoma and leiomyosarcoma which is similar to what has been reported in the literature before [2]. Osteochondromas made up 17% (*n* = 71) of all our cases which is lower than the proportion found in a similar study [8]. Osteochondromas were predominantly found in the first four decades of life (*n* = 61) and therefore the difference in proportion may reflect the differences in age between the two cohorts (Figure 5).

Bones are commonly affected by metastatic cancer and metastases are the most common cause of malignant bone lesions [11]. Breast and prostate are the most common malignancies to disseminate to bone and these predominantly affect the axial skeleton, however the scapula can also be affected [11]. Our analysis of scapula metastases found lung, renal and adenocarcinoma of unknown origin to be the most common primary sites.

### Conclusions

Our analysis of this large series has highlighted certain features of the epidemiology of scapula lesions. We found that more than half of all the lesions are malignant and the rate of malignant lesions increases with increasing age. A wide variety of malignant and benign conditions can affect the scapula and this along with the difficult location and peculiar anatomy of this bone can make diagnosis challenging. We recommend a low threshold for imaging and biopsy of suspicious lesion of the scapula and recommend that they should be dealt with in a specialist sarcoma unit in order to receive the correct systemic diagnostic work up in a multidisciplinary team setting.

### Limitations

Retrospective study over a 30-year period.

## Conflict of interest

The author(s) declare no conflict of interest in relation with this paper.
